# Noninvasive Intelligent Brain‐Metasurface System for Real‐Time Electromagnetic Wave Manipulation

**DOI:** 10.1002/nap2.70194

**Published:** 2026-07-03

**Authors:** Xiao Hai, Ruiyang Shen, Xiaoyi Wang, Guo‐Min Yang

**Affiliations:** ^1^ Key Laboratory for Information Science of Electromagnetic Waves School of Information Science and Technology Fudan University Shanghai China; ^2^ College of Electronic and Information Engineering Tongji University Shanghai China

**Keywords:** BCI, CNN, metasurfaces, motor imagery

## Abstract

A noninvasive intelligent brain–computer interface (BCI) is integrated with a reconfigurable metasurface to establish a direct neural‐to‐electromagnetic transduction pathway for real‐time, multimodal wave control. By combining convolutional‐neural‐network‐based decoding of motor‐imagery electroencephalography (EEG) signals with threshold‐based recognition of electrooculography (EOG) commands, the proposed system converts neural and ocular activities into high‐speed control commands, which are subsequently implemented through an FPGA‐driven dual‐polarized reconfigurable metasurface for real‐time electromagnetic‐wave manipulation. This architecture concurrently enables beam steering, polarization conversion, and radar cross‐section (RCS) reduction within a unified platform. Three distinct operational modes are demonstrated: polarization‐invariant beam deflection, joint steering and polarization conversion, and broadband RCS reduction via random phase coding. Experimental results reveal a peak gain of 18.54–19.29 dBi with a 3 dB beamwidth of 12°–15° at a +20° steering angle, and an RCS reduction exceeding 10 dB across 3.7–3.85 GHz. The proposed intelligent BCI‐metasurface paradigm validates the feasibility of brain‐controlled electromagnetic manipulation, opening new avenues for adaptive wireless communications, immersive virtual environments, and next‐generation assistive technologies.

## Graphical abstract

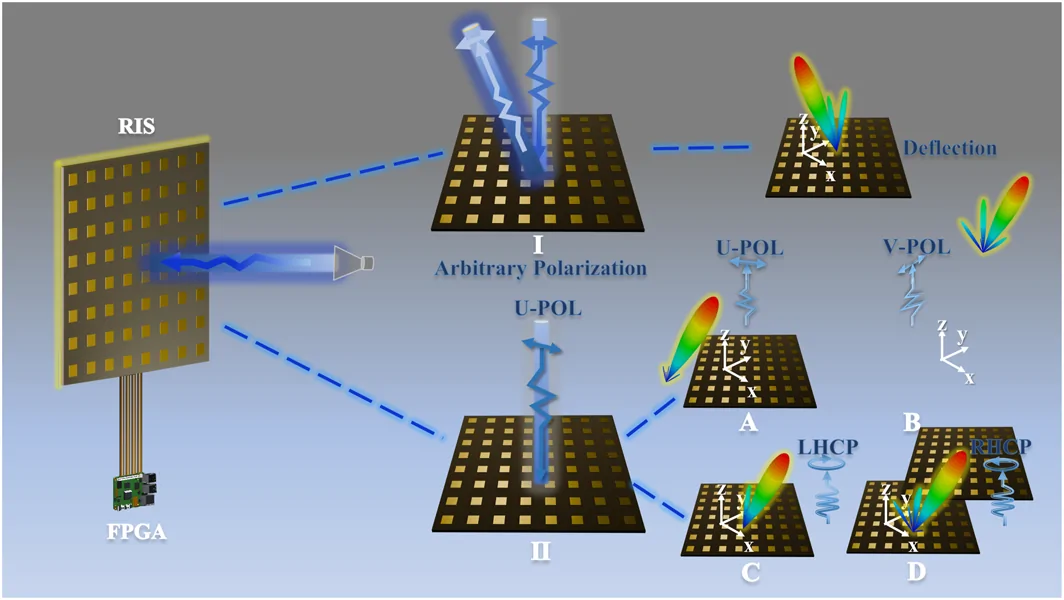

## 1 Introduction

Noninvasive brain–computer interface (BCI) has emerged as a powerful paradigm for translating human intent into machine commands, enabling direct neural control of external devices without surgical intervention. This capability holds profound implications for assistive technologies, immersive virtual environments, and adaptive human–machine collaboration. Extending BCI control to the manipulation of electromagnetic (EM) waves in real time—an as‐yet largely unrealized capability—would open unprecedented avenues for neural‐driven wireless communications, adaptive sensing, and reconfigurable intelligent surfaces.

Among noninvasive BCI modalities, electroencephalography (EEG) remains the most widely adopted owing to its portability, temporal resolution, and established decoding frameworks. Motor imagery (MI) paradigms, in particular, offer intuitive, endogenous control by decoding sensorimotor rhythms without requiring external stimuli [1, 2, 3, 4, 5]. However, MI decoding faces persistent challenges including intersubject variability and limited class separability. Recent advances in compact neural networks, cross‐subject transfer learning, and hybrid designs that fuse EEG with electrooculography (EOG) have substantially improved decoding robustness and expanded the viable command vocabulary [6, 7, 8, 9, 10]. Such hybrid BCIs provide reliable, multichannel intent signals suitable for real‐time control applications [11, 12, 13, 14].

Parallel to these developments, metamaterials have evolved from static, bulk composites with fixed EM responses into digitally coded and time‐variant metasurfaces capable of dynamic wavefront engineering. Space–time‐coding metasurfaces further extend programmability to the spectral and nonreciprocal domains, enriching the degrees of freedom available to system designers [15, 16]. Coding programmable metasurface frameworks discretize the aperture phase or amplitude distribution, enabling agile beam steering, polarization conversion, and radar cross section (RCS) modulation under electronic control [17, 18, 19, 20, 21, 22, 23]. Despite these advances, most programmable metasurfaces remain tethered to predefined or sensor‐driven switching logics; direct, real‐time neural command of their EM responses has only recently been explored.

The recent convergence of BCIs with programmable metasurfaces has produced several proof‐of‐concept systems. Noninvasive EEG has been utilized for direct wireless communication and device control through information metasurfaces and STC platforms [24, 25]. Brain–computer‐metasurface holography has demonstrated neural‐to‐wavefront conversion with scene‐level reconfigurability [26], whereas brainwave and voice‐interactive metasurfaces have showcased multimodal intent mapping and bidirectional human and EM interactions [27, 28]. Despite these advances, most reported systems still rely on exogenous evoked potentials, discrete command sets, single‐polarization operation, or limited functional reconfigurability, thus constraining functional density and adaptability in complex electromagnetic environments. A unified platform that simultaneously supports multiple high‐level EM functionalities under endogenous, hybrid neural control is yet to be demonstrated.

Reconfigurable metasurfaces provide a mature and versatile hardware foundation to address these challenges. Extensive studies have quantified phase‐quantization effects and enabled electronically reconfigurable unit cells with 1‐bit to 2‐bit resolution [29, 30, 31, 32]. Existing designs support independent control of linear or circular polarization, dual‐band and dual‐channel responses, and spin‐decoupled operations, offering scalable routes toward multifunctional apertures [33, 34, 35, 36, 37]. Recent reviews and representative demonstrations of reconfigurable intelligent surfaces and multifunctional metasurfaces have further highlighted their programmability, implementation strategies, and practical applications in electromagnetic‐wave manipulation and wireless systems [38, 39]. Recent developments in STC metasurfaces further span near‐field focusing with multifocus control, secure wireless communication via time coding, real‐time 2‐bit operations, and oriented application‐specific prototypes, forming a consistent theory to hardware research chain [40, 41, 42, 43, 44, 45]. These capabilities align naturally with intent mapping in BCI, where a compact set of reliable neural and oculographic commands can select operation modes and dynamically adjust beam and polarization states in real time.

Motivated by these developments, this study integrates an EEG/EOG‐based hybrid BCI with a dual‐polarization, 2‐bit reconfigurable reflective metasurface (RRM), demonstrating three intent addressable operation modes as shown in Figure 1. EEG and EOG signals are acquired through the OpenBCI CytonDaisy biosensing board. A pretrained convolutional neural network (CNN) is used to recognize the user's intent signals, enabling dynamic modulation of the metasurface for electromagnetic wave control. The proposed multiscale filter bank convolutional neural network (MSFBCNN) offers several advantages for EEG‐based motor imagery decoding. The incorporation of multiscale temporal filters enhances the receptive field, enabling robust extraction of frequency‐domain features across multiple bands. Nonlinear activation functions based on square and logarithmic operations further improve sensitivity to band‐power variations associated with motor intentions. Moreover, the integration of batch normalization and dropout regularization mitigates overfitting under limited training data. Collectively, these features provide a compact yet expressive model that achieves high classification accuracy, fast convergence, and strong generalization in intent recognition tasks. In the metasurface design, a dual‐polarization and independently tunable 2‐bit reflective unit cell is proposed, and an 8 × 8 space‐fed metasurface is constructed accordingly. Each unit cell integrates two orthogonal polarization control channels, enabling independent phase manipulation along the *x*‐ and *y*‐polarization. By applying discrete bias voltages to the PIN‐diode networks, four distinct reflection‐phase states of 0°, 90°, 180°, and 270° are synthesized per polarization, achieving 2‐bit modulation. The 8 × 8 metasurface array is excited through a horn feed, and the spatial phase distribution across the aperture is programmed to realize different beam steering and polarization conversion functionalities.

**FIGURE 1:**
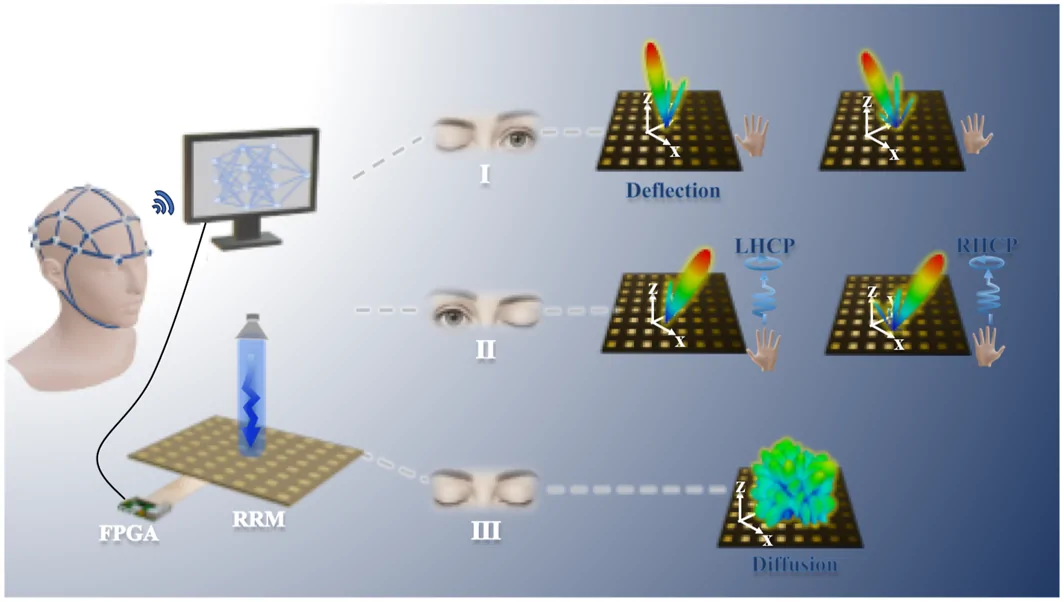
Schematic diagram of the proposed BCI‐controlled reconfigurable metasurface system.

The platform integrates a noninvasive BCI, a field programmable gate array (FPGA), a computer, and a RRM to manipulate electromagnetic waves in real time. In this system, EEG‐based MI signals are used to drive the primary metasurface control modes, whereas EOG signals serve as auxiliary inputs for mode switching. Hybrid EOG and EEG signals are decoded and mapped to three modes. Mode‐I represents beam deflection in which the reflected beam is steered toward specific directions. Mode‐II performs joint control that combines beam steering and polarization conversion, where left‐hand and right‐hand motor imagery correspond to +20° beam steering with LHCP and RHCP conversion, respectively. Mode‐III corresponds to the function of RCS reduction, which can be achieved by the proposed RRM by properly arranging the phase response distribution randomly. These modes demonstrate intent‐driven, real‐time control of complex EM functions. Such integration bridges neural intelligence with programmable electromagnetic control, enabling low latency translation from neural signals to multifunctional EM responses. This proposed system solution highlights the application potential of robust brain–computer interfaces with electromagnetic equipments in the fields of assistive control, immersive environments, and adaptive wireless links, marking a crucial step toward next‐generation neural electromagnetic sensing and interaction systems.

## 2 BCI

### 2.1 Signal Acquisition and Data Description

The low‐cost OpenBCI CytonDaisy biosensing board is employed to acquire EEG and EOG signals, featuring a 32‐bit processor and 16 input channels. All signals are sampled at 1000 Hz and transmitted wirelessly via a Wi‐Fi module. As shown in Figure 2a, electrodes are positioned around the sensorimotor cortex, including C3, C4, Cz, CP3, CP4, FC3, and FC4, whereas Fp1 and Fp2 are used to record EOG signals. Figure 2b presents the timeline of a single trial. Each trial begins with a 2 s fixation cross, followed by an auditory cue and a directional arrow indicating the MI task. Participants are instructed to imagine corresponding hand movements during a 2 s window. Visual feedback is then displayed to show the system's decoded result, followed by a 1 s black screen before the next trial begins.

**FIGURE 2:**
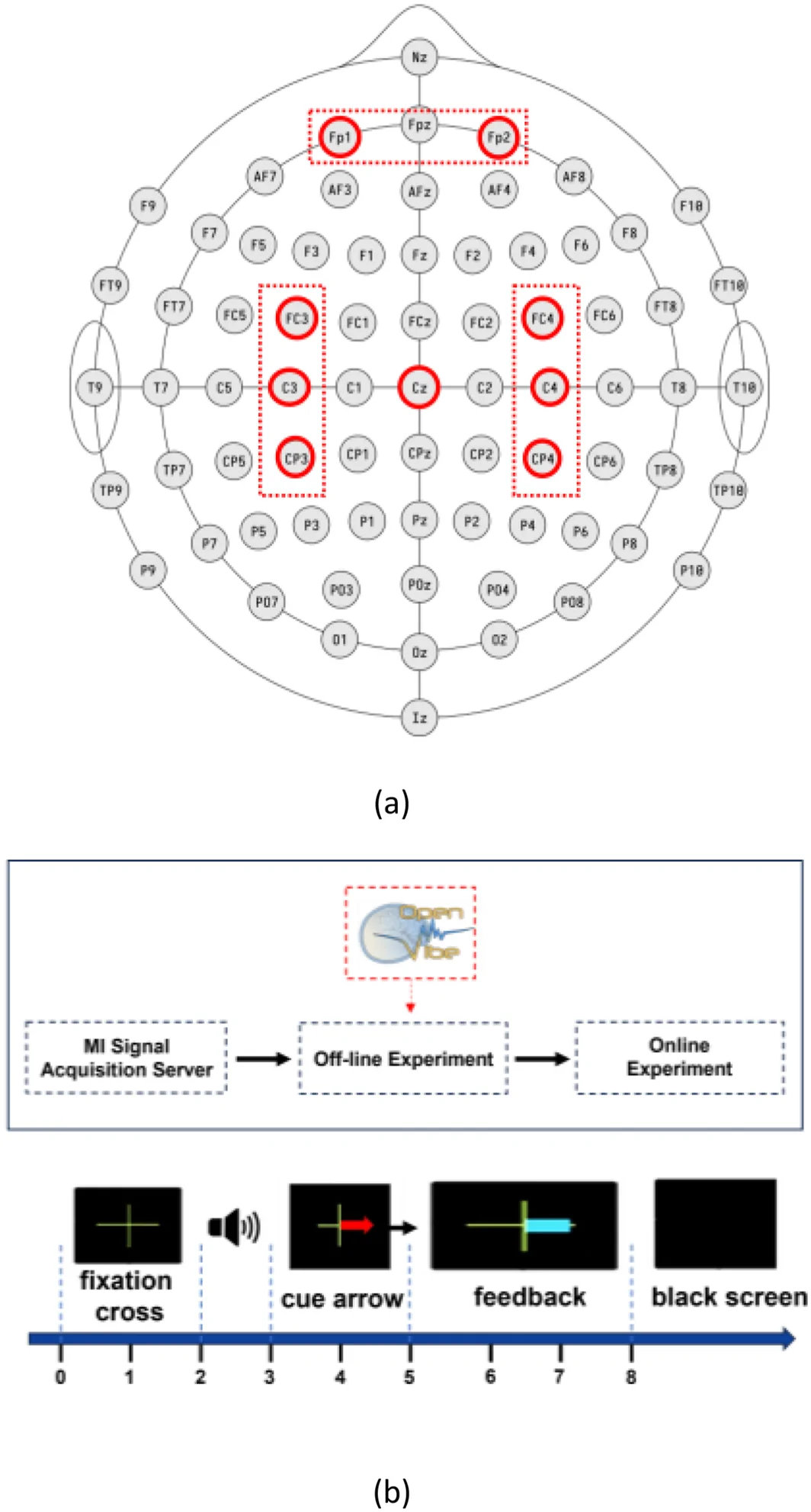
(a) Electrode placement map for EEG and EOG signal acquisition. (b) Timeline of a single trial during the signal acquisition process.

This procedure ensures consistent timing and reduces cognitive interference. The reference electrode is placed on the earlobe, and all signals are recorded using the OpenViBE open‐source BCI soFtware [11]. For BCI calibration, EEG data corresponding to both rest and MI states were collected from each participant for BCI calibration. EEG data corresponding to two states were collected from each participant across seven sessions. Each session contained 12 trials per class. Six sessions were used for training, yielding 72 training trials per class, whereas the remaining session provided 12 test trials per class. During data acquisition, the participant was seated comfortably approximately 70 cm in front of a laptop monitor and wore a 16‐channel EEG cap connected to an OpenBCI Cyton‐Daisy amplifier, as shown in Figure 3. Visual cues in the form of left‐ or right‐pointing arrows were presented at the center of the screen to instruct the corresponding hand motor imagery.

**FIGURE 3:**
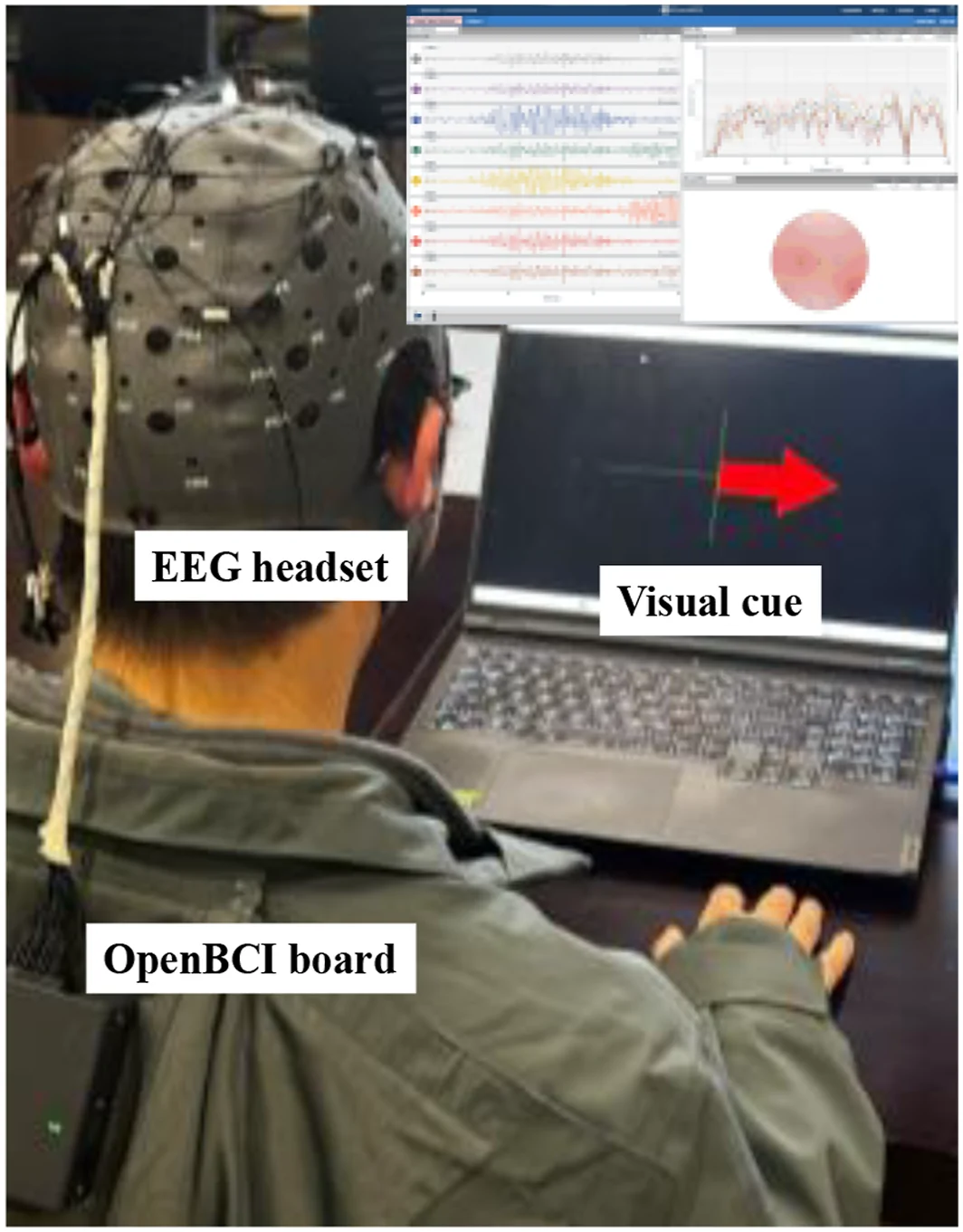
Experimental setup for motor imagery EEG acquisition.

### 2.2 Signals Processing

To enable switching among the three primary metasurface modes using EOG signals, participants were instructed to perform three types of intentional eye blinks: left‐eye blink, right‐eye blink, and simultaneous blink of both eyes. Intended blinks generally exhibit higher amplitudes than unintentional ones due to stronger ocular motion. Thus, a simple threshold detection algorithm was implemented for discrimination. Specifically, a 600 ms EOG epoch was analyzed from the onset of each command to determine whether it contained an intended blink. A detected waveform was classified as intentional if it occurred within a predefined temporal window following the command onset and exceeded a preset amplitude threshold. In the current implementation, EEG and EOG signals are processed through separate decoding pathways. MI–EEG signals are classified by the proposed MSFBCNN, whereas EOG commands are recognized using a threshold‐based detector. Although a unified multimodal decoding framework is feasible, the present design adopts separate processing streams to reduce computational complexity and ensure robust mode switching.

For EEG‐based MI recognition, a layered convolutional neural network was developed, consisting of a feature extraction network embedded into an end‐to‐end framework. EEG signals were recorded from eight electrodes at a sampling rate of 1000 Hz, forming an input matrix of 8 × 1000 per trial. The proposed MSFBCNN comprises three main components: a feature extraction layer, a regularization layer, and a classification layer.

Because EEG features are distributed across both temporal and spatial domains, separable 2‐D convolutions with kernel sizes [*k*, 1] and [1, *C*] were used to extract temporal and spatial features, respectively. A parallel multiscale temporal filter bank was designed to enhance feature extraction performance, where the filter length was manually tuned according to the signal characteristics and sampling frequency. Extracted temporal features were batch‐normalized and then fed into the spatial convolutional layer to reduce channel dimensionality.

Nonlinear square and logarithmic activation functions were introduced to enhance the network's capacity to capture band‐power‐related features, followed by an average‐pooling layer and a dropout layer (*p* = 0.5) to prevent overfitting. The final classification was performed by a fully connected layer, which outputs the predicted user intent.

As shown in Figure 4, the MSFBCNN employs multiscale temporal convolutions with kernel lengths of 64, 40, 26, and 16, whose outputs are concatenated before spatial convolution to reinforce the extraction of frequency‐domain features. The length of the spatial filter equals the number of EEG channels. This design improves the temporal receptive field and enhances the robustness of the network under limited training data. Detailed architectural parameters are summarized in Table 1, where *T* denotes time points, C the number of channels, F_*T*_ and F_*S*_ the temporal and spatial filters, *D* the ratio between F_*T*_ and F_*S*_, and *NC* the number of classes.

**FIGURE 4:**
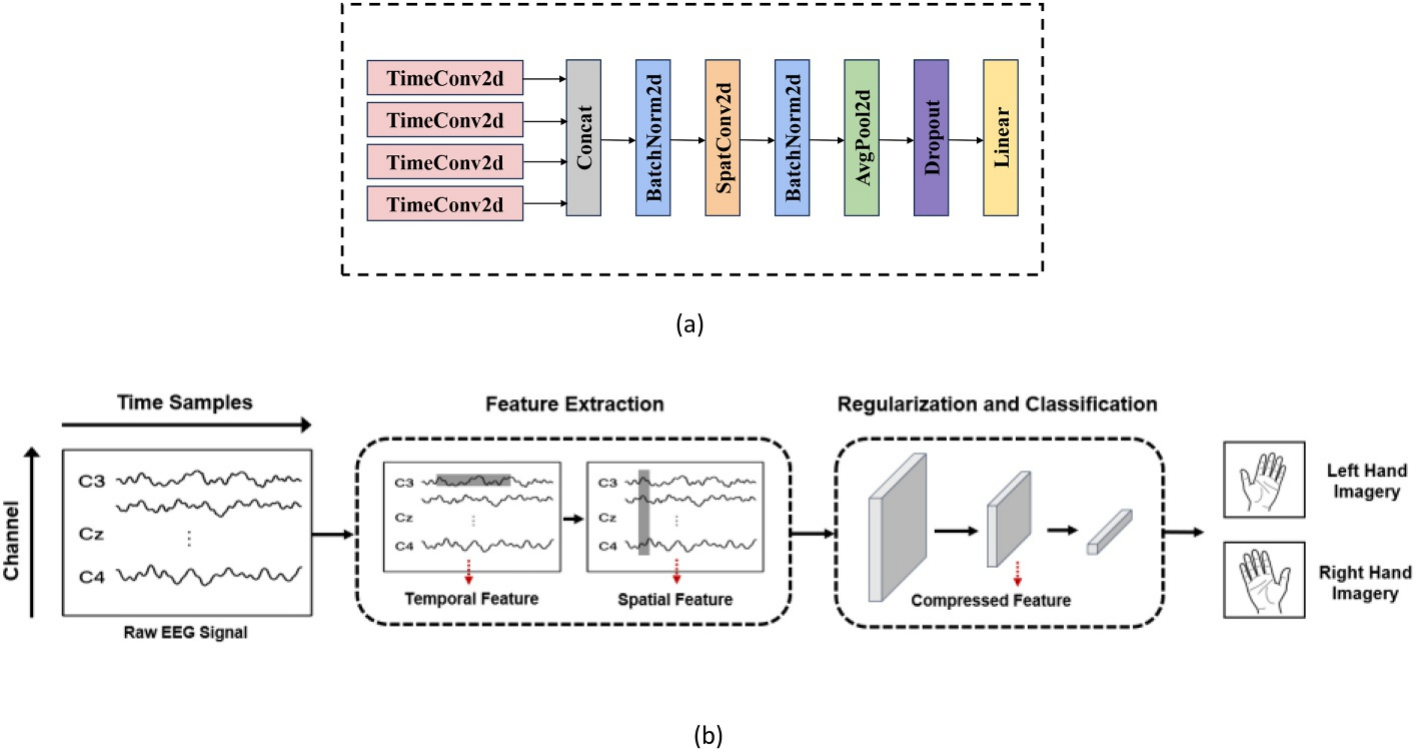
Overall architecture of the proposed neural network for MI classification tasks. (a) Schematic of the MSFBCNN architecture. (b) Schematic of the MI–EEG decoding pipeline.

**TABLE 1:** Detailed architecture of the proposed network.

Layer	# Filters	Kernel	Stride	# Params	Output	Activation	Padding
Input					(*C, T*)		
Reshape					(1, *T, C*)		
TimeConv1	*F_T_*	(64, 1)	(1, 1)	64 *F_T_*	(*F_T_*, *T, C*)	Linear	Same
TimeConv2	*F_T_*	(40, 1)	(1, 1)	40 *F_T_*	(*F_T_*, *T, C*)	Linear	Same
TimeConv3	*F_T_*	(26, 1)	(1, 1)	26 *F_T_*	(*F_T_*, *T, C*)	Linear	Same
TimeConv4	*F_T_*	(16, 1)	(1, 1)	16 *F_T_*	(*F_T_*, *T, C*)	Linear	Same
Concat					(4 *F_T_*, *T, C*)		
BatchNorm				2 *F_T_*	(4 *F_T_*, *T, C*)		
SpatialConv	*F_S_*	(1, *C*)	(1, 1)	*C* 4 *F_T_ F_S_*	(*F_S_*, *T, 1*)	Linear	Valid
BatchNorm				2 *F_S_*	(*F_S_*, *T, 1*)		
Non‐linear					(*F_S_*, *T, 1*)	Square	
AveragePool		(75, 1)	(1, 1)		(*F_S_*, *T*//15, 1)		Valid
Non‐linear						Log	
Dropout					(*F_S_*, *T*//15, 1)		
Classifier	*NC*	(*T*//15, 1)	(1, 1)	*F_S_* (*T*//15) *NC*		Linear	Valid

The MSFBCNN is selected as the decoding backbone because it matches the statistical structure of motor imagery EEG while remaining compact enough for real‐time BCI‐based metasurface control. First, the network operates directly on band‐limited time‐domain signals and integrates feature extraction and classification in an end‐to‐end manner, avoiding hand‐craFted features such as Common Spatial Pattern (CSP) or manually designed frequency bands, thus, reducing engineer‐dependent bias. Second, the multiscale temporal convolutions can be interpreted as a learnable filter bank: the four kernel lengths (64, 40, 26, and 16) provide different temporal receptive fields that cover *μ*/*β* oscillations in the 8–30 Hz range and their subject‐specific variations, improving robustness to frequency shiFts and allowing reliable decoding from short 1 s MI segments. Third, separable two‐dimensional convolutions decouple temporal and spatial filtering, which substantially reduces the number of trainable parameters compared with full two‐dimensional convolutions, while still capturing discriminative spatial patterns over C3/Cz/C4 and neighboring channels. This property is particularly advantageous under the limited sample conditions typical of MI experiments.

In addition, the use of square and logarithmic nonlinearities emphasizes band‐power related features that are known to carry event‐related desynchronization/synchronization (ERDS) information, thereby establishing a clear connection between classical power‐spectrum features and deep learning representations. Combined with average pooling and dropout, the resulting architecture achieves a favorable balance between classification performance and regularization, enabling stable training without overfitting. Compared with more computationally intensive RNN‐ or Transformer‐based models, the proposed MSFBCNN has a substantially smaller computational footprint and lower inference latency, making it suitable for online operation on standard computing platforms. These properties make the MSFBCNN particularly appropriate for our application scenario, in which reliable decoding must be achieved from short MI segments and subsequently mapped to low‐latency, feedforward control commands for the 2‐bit reconfigurable metasurface.

### 2.3 Results Analysis

To evaluate the reliability of the EOG‐based mode‐switching mechanism, a continuous operation test was conducted. For each blink command, a 600 ms EOG epoch was extracted from the Fp1 and Fp2 channels. The recorded signals were first filtered and differentiated to enhance blink‐related transient features. A multithreshold detection strategy was then applied, including amplitude, positive/negative peak, and duration constraints. A blink command was accepted only when the waveform exceeded the subject‐specific threshold and satisfied the predefined temporal criteria within the 600 ms window.

EOG amplitudes associated with intentional eye movements are typically below 500 μV and are correlated with eye‐movement magnitude. Small‐amplitude eye movements are more susceptible to DC driFt and noise contamination. Therefore, intentional blink commands with relatively large amplitudes and short durations were adopted in this work to improve recognition reliability.

A total of 500 blink‐command trials were evaluated during continuous operation. Among these trials, 482 resulted in correct mode switching, corresponding to an overall accuracy of 96.4%. The false‐trigger and missed‐trigger rates were 2.2% and 1.4%, respectively. These results demonstrate that the proposed EOG‐based mode‐selection mechanism provides reliable and robust operation for the hybrid EOG–EEG control framework.

Figure 5a presents representative time‐domain EEG traces recorded from eight channels during the motor imagery task. The signals exhibit clear neural oscillatory activity with amplitudes on the order of tens of microvolts, which is consistent with typical scalp EEG recordings. Across channels, no abnormal saturation or clipping is observed, indicating stable signal acquisition throughout the recording period. Moreover, noticeable temporal modulations in signal amplitude can be observed during the task interval, suggesting task‐related neural engagement. These observations confirm that the acquired EEG signals possess sufficient signal quality and dynamic variability to support subsequent frequency‐domain analysis and motor imagery decoding.

**FIGURE 5:**
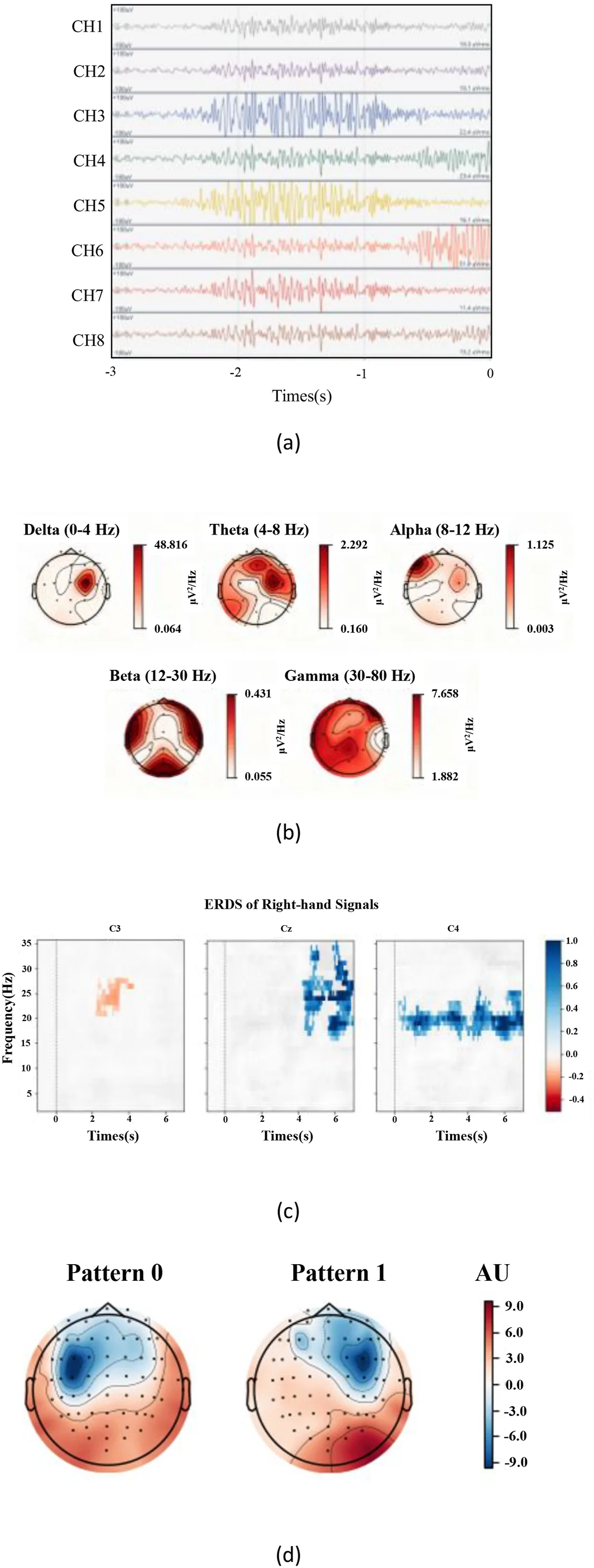
EEG signal validity analysis for motor imagery decoding. (a) Waveforms of the eight‐channel EEG signals. (b) Scalp topographies of band‐limited power spectral density (μV^2^/Hz) in the delta (0–4 Hz), theta (4–8 Hz), alpha (8–12 Hz), beta (12–30 Hz), and gamma (30–80 Hz) bands. (c) ERD/ERS time–frequency maps at C3, Cz, and C4 for right‐hand motor imagery. (d) CSP spatial patterns for left‐versus right‐hand MI EEG.

Figure 5b illustrates the scalp distributions of power spectral density (PSD) across five canonical EEG frequency bands, including delta (0–4 Hz), theta (4–8 Hz), alpha (8–12 Hz), beta (12–30 Hz), and gamma (30–80 Hz). These topographic maps are presented to assess the physiological plausibility of the recorded EEG signals rather than to directly optimize classification performance. As expected, relatively pronounced spectral power is observed over central and sensorimotor regions in the alpha and beta bands, which are known to be closely associated with motor imagery processes. In particular, spatially structured power modulation around the central electrodes (C3, Cz, and C4) indicates the presence of rhythmical sensorimotor activity. The remaining frequency bands exhibit distinct but less task‐specific spatial patterns, reflecting background neural activity and broadband cortical dynamics. Overall, the observed PSD distributions are consistent with established neurophysiological characteristics of motor imagery EEG signals, supporting the validity of the acquired data for time–frequency analysis and decoding.

Figure 5c presents the ERDS time–frequency representations of EEG signals recorded at electrodes C3, Cz, and C4 during right‐hand motor imagery. The color scale represents task‐related power modulation relative to the baseline, where red indicates event‐related desynchronization (ERD) and blue event‐related synchronization (ERS). Clear task‐related spectral modulation is observed in the beta frequency range following task onset. Specifically, electrode C3, located over the left sensorimotor cortex contralateral to the imagined right‐hand movement, exhibits discernible beta‐band ERD, reflecting task‐induced cortical activation. In contrast, the C4 electrode exhibited relatively enhanced blue‐zone synchronization in the beta band, indicating inhibitory activity in the ipsilateral sensorimotor cortex. These spatially and temporally structured ERDS patterns are consistent with the established neurophysiological mechanisms of motor imagery, demonstrating contralateral desynchronization and region‐specific spectral reorganization. The presence of such characteristic ERDS signatures confirms that the recorded EEG signals contain discriminative motor imagery‐related information suitable for subsequent decoding and classification.

Figure 5d illustrates the scalp topographies of the first two CSP components derived from the binary motor imagery classification task. In Pattern 0, a pronounced negative weighting is observed over the left central sensorimotor region, approximately corresponding to electrode C3, whereas strong positive contributions are distributed over the inferior and contralateral scalp areas. This asymmetric spatial distribution reflects a canonical contralateral sensorimotor activation pattern associated with right‐hand motor imagery. The dominance of discriminative weights over the left hemisphere indicates that this CSP component primarily captures neural activity related to right‐hand MI, consistent with the contralateral organization of the motor cortex.

As shown in Figure 6a, the cross‐subject MI EEG classification performance was evaluated using a leave‐one‐subject‐out (LOSO) protocol across five subjects (S1‐S5). The subject‐wise accuracies were consistently distributed within approximately 68%–74%, and all subjects achieved performance clearly above the chance level of 50%. The overall mean accuracy is indicated by the horizontal line, while the per‐run variability for each subject is visualized by the scatter points and error bars. Figure 6b presents the confusion matrix of the cross‐subject LOSO evaluation. The diagonal entries indicate correct classification rates of 75.6% for the Left class and 77.3% for the Right class, whereas the off‐diagonal entries correspond to misclassifications between the two MI tasks. Overall, the confusion matrix suggests a relatively balanced recognition performance for left‐ and right‐hand motor imagery, with comparable class‐wise accuracies. The moderate cross‐subject accuracy is mainly driven by three factors. First, sensorimotor rhythms vary substantially across individuals, including differences in peak frequencies and spatial distributions, making class‐discriminative patterns less consistent across subjects. Second, recording and session conditions introduce additional variability, such as changes in electrode placement and impedance, ocular artifacts, muscle activity, and fluctuations in attention. Third, left versus right motor imagery has inherently limited separability, and the training data are relatively limited, so the model tends to learn subject‐specific signatures rather than robust subject‐invariant representations.

**FIGURE 6:**
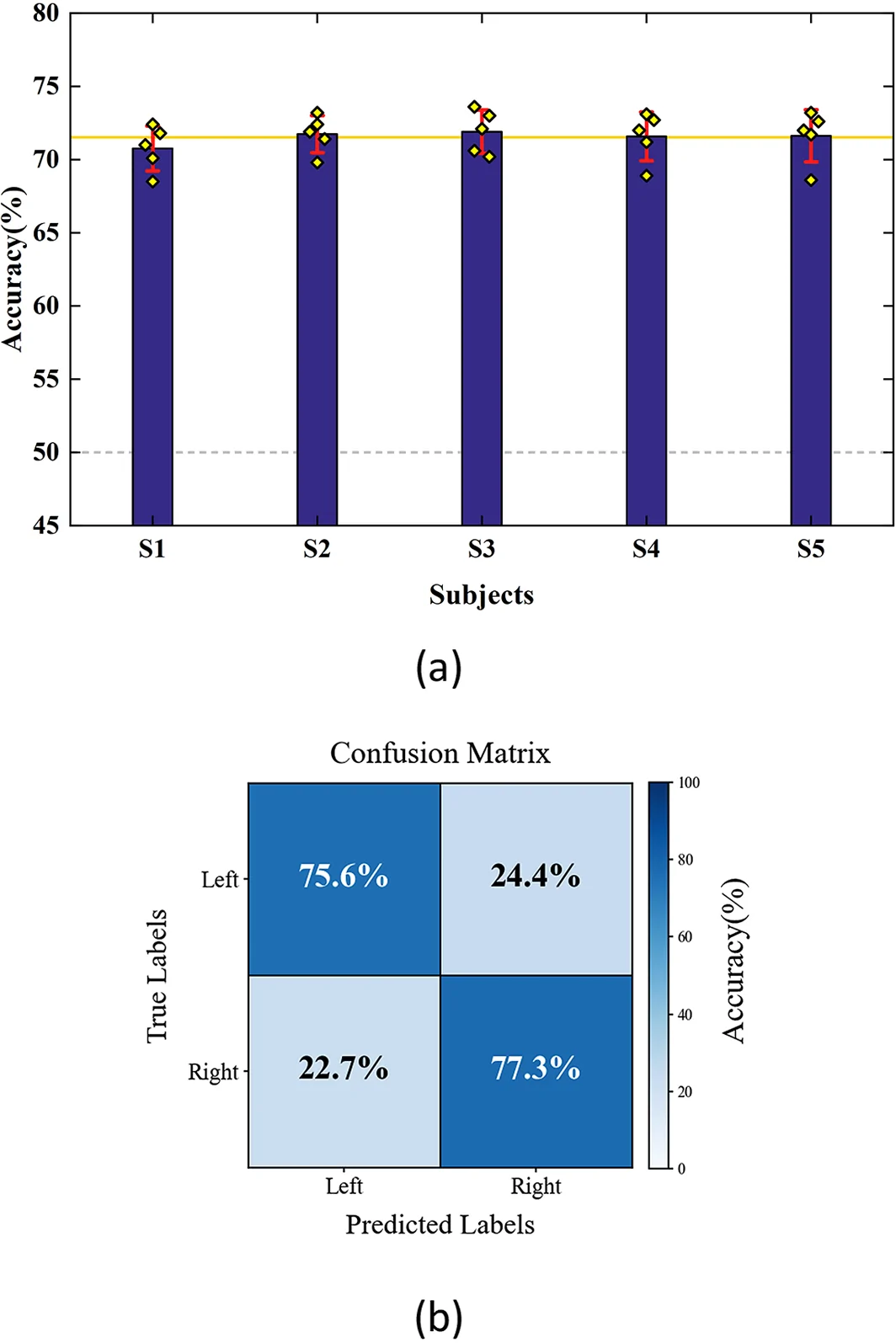
Decoding performance of the MSFBCNN. (a) Cross‐subject MI EEG classification performance under the LOSO protocol. (b) Confusion matrix of cross‐subject MI EEG classification.

## 3 Design of the 2‐Bit Reconfigurable Metasurface

### 3.1 Unit Cell Design

The RRM element is depicted in Figure 7. As shown in Figure 7a, the unit cell adopts a multilayer stack comprising three patterned metal layers separated by two F4b substrates with relative permittivity = 2.47 and loss tangent tan = 0.002. The substrate thicknesses are *h*1 = 3 mm and *h*2 = 1 mm. As shown in Figure 7b, the top metallization on Substrate 1 is a radiating patch that couples to free space. It serves as the aperture interface for both polarizations. In Figure 7c, the bottom metallization on the underside of Substrate 2 integrates two identical reflective phase shiFters, enabling independent 2‐bit reflection phase control along the *x*‐ and *y*‐polarization components. Each reflective phase shiFter consists of a microstrip transmission line loaded by a pair of vertical stubs, a sector‐shaped open stub, and a horizontal stub. PIN diodes are used to realize discrete state switching: PIN1 and PIN2 bridge between the horizontal stub and the two vertical stubs, respectively, whereas PIN3 connects the main line to the sector shaped open stub. By appropriate biasing of the three diodes, four discrete reflection‐phase states are synthesized per axis. An intermediate continuous ground plane, sandwiched between Substrate 1 and Substrate 2, is shared by the top patch and the lower reflective networks, acting simultaneously as the RF ground and as an EM shield that separates the radiating aperture from the DC bias routing, thereby mitigating parasitic scattering from dense bias lines in array implementations. As shown in Figure 7d, two plated through vias interconnect the top patch to two reflective phase shiFters, providing independent control of the reflected phase for *y*‐ and *x*‐polarized incidence. To avoid shorting to the ground plane, circular clearance holes of radius 1 mm are opened in the intermediate ground at the locations of Via 1 and Via 2.

**FIGURE 7:**
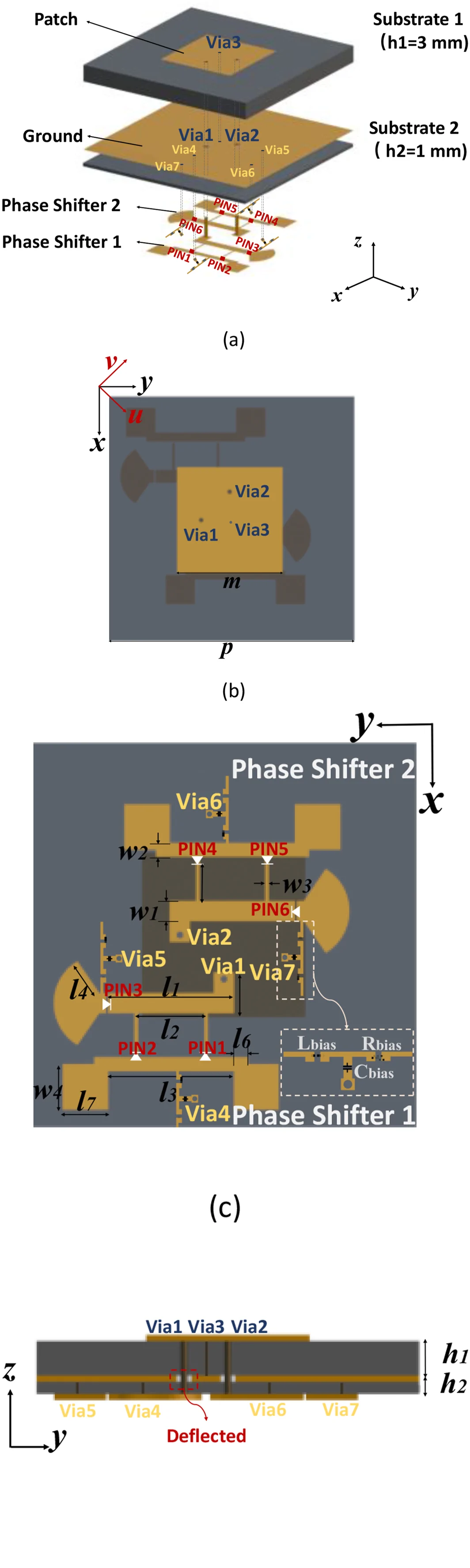
Geometry of the proposed dual‐polarization 2‐bit reconfigurable metasurface element. (a) Exploded view. (b) Top view. (c) Bottom view. (d) Side view. Detailed dimensions: *p* = 55 mm, *m* = 23 mm, *d*_1_ = 4 mm, *d*_2_ = 4 mm, *l*_1_ = 18 mm, *l*_2_ = 9.5 mm, *l*_3_ = 14 mm, *l*_4_ = 6 mm, *l*_5_ = 1 mm, *l*_6_ = 2 mm, *l*_7_ = 6.5 mm, *w*_1_ = 2.88 mm, *w*_2_ = 2 mm, *w*_3_ = 0.5 mm, *h*_1_ = 3 mm, *h*_2_ = 1 mm.

Full‐wave simulations of the metasurface unit cell were performed in CST Studio Suite. To emulate an infinite periodic array, Unit Cell boundary conditions were applied and the structure was excited by Floquet ports under plane wave illumination. The PIN diode MADP‐000907‐14020 was modeled by an equivalent circuit. In the ON state, a series resistance and inductance were used, whereas in the OFF state, a series capacitance and inductance were adopted. The parameter values were *R*_*on*_ = 2 Ω, *L*_*on*_ = 0.1 *nH*, *C*_*off*_ = 0.020 *pF*, *L*_*off*_ = 0.1 nH. The resulting reflection responses were extracted under these settings for unit cell characterization and codebook synthesis.

A 2‐bit reflective phase shiFter (RPS) is employed with four digital states: “00,” “01,” “10,” and “11.” Each code is a 2‐bit word (b1b0). Here, b1denotes the bias state of the pair of PIN diodes between the main transmission line and the vertical stub, and b0 denotes the bias state of the PIN diode between the main line and the fan‐shaped open stub. A “0” corresponds to the reverse‐biased (OFF) state, and a “1” corresponds to the forward‐biased (ON) state. To distinguish the two orthogonal polarization channels, *x* and *y* denote the codes applied to RPS‐2 (*x*‐polarization) and RPS‐1 (*y*‐polarization), respectively. Accordingly, the *x*‐polarization governs the reflected phase for *x*‐polarized incidence and the *y*‐polarization governs that for *y*‐polarized incidence. The correspondence between the specific unit cell codes and the PIN diode states is listed in Table 2.

**TABLE 2:** Relationship between reflected polarization/phase and orthogonal 2‐bit phase states.

States	PIN4/5	PIN6	PIN1/2	PIN3
x00y00	OFF	OFF	OFF	OFF
x00y01				ON
x00y10			ON	OFF
x00y11				ON
x01y00		ON	OFF	OFF
x01y01				ON
x01y10			ON	OFF
x01y11				ON
x10y00	ON	OFF	OFF	OFF
x10y01				ON
x10y10			ON	OFF
x10y11				ON
x11y00		ON	OFF	OFF
x11y01				ON
x11y10			ON	OFF
x11y11				ON

Full‐wave simulation results under different polarizations are summarized in Figure 8. As shown in Figure 8a, at the center frequency of 3.75 GHz with a linear polarization angle of *α* = 0° (defined with respect to the *x*‐axis), the reflected phases for the four codes x00y00, x10y10, x01y01, and x11y11 are −92°, 0°, 90° and 167°, respectively, exhibiting an approximately 90° progression between adjacent states. Over the 3.70–3.80 GHz operating band, when evaluated for linear polarization angles *α* = 0°, 30°, 60°, 120°, the code invariant reflected phases are essentially maintained, thereby realizing stable 2‐bit phase quantization. The small discrepancies in phase for the same code under different incident polarizations are attributed to minor mismatches in the amplitude and phase responses of the unit cell along the orthogonal *x*‐ and *y*‐polarized waves. As shown in Figure 8b, the reflected magnitude remains generally flat across the operating band. Small dips are mainly caused by slight amplitude mismatches between the responses along two orthogonal directions and local resonances, but the overall magnitude is consistent, satisfying a phase‐dominant control design.

**FIGURE 8:**
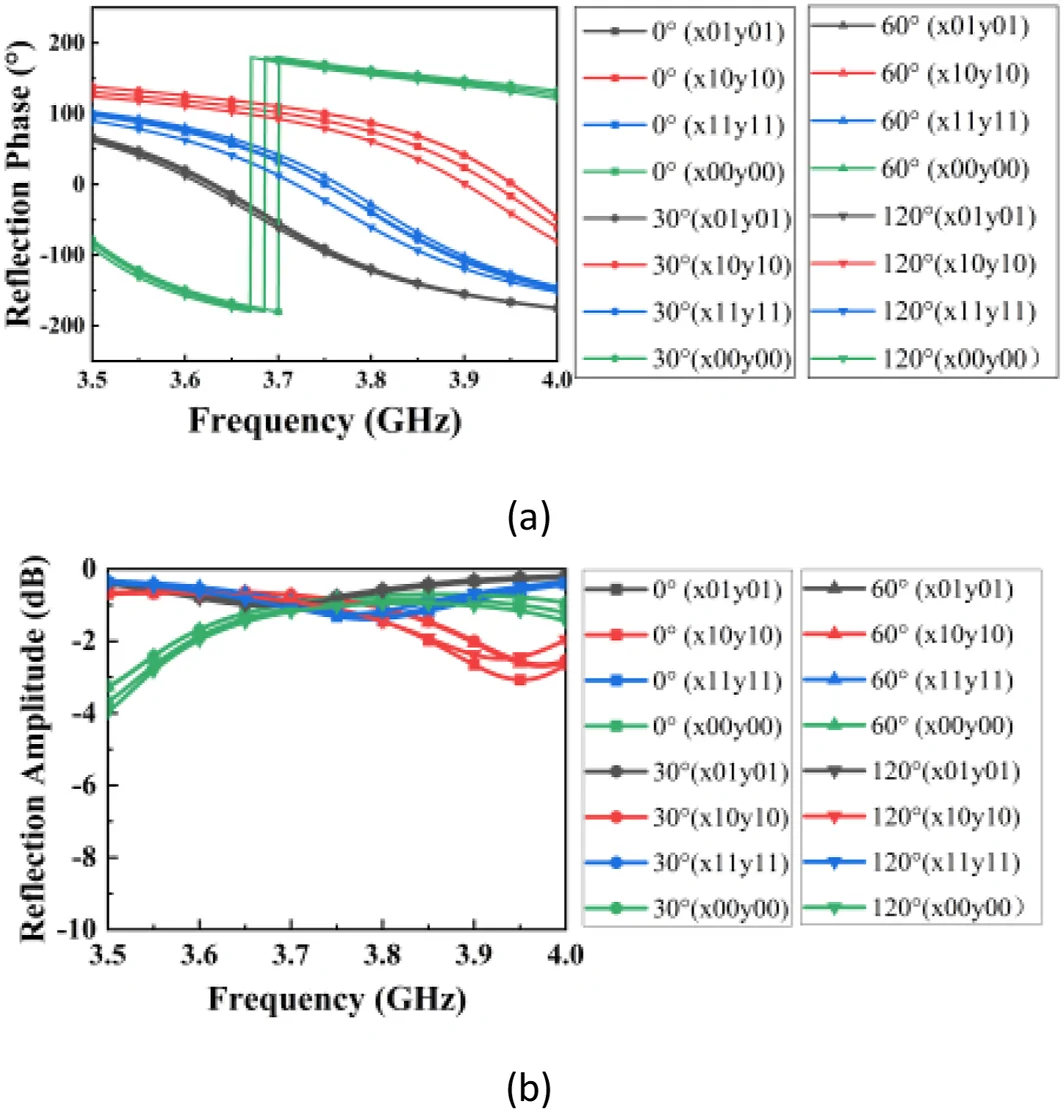
Simulated responses of the metasurface unit cell under linearly polarized incidence at various polarization angles *α*. (a) Reflected phase. (b) Reflected magnitude.

Under *u*‐polarized incidence, the unit cell enables simultaneous control of polarization and phase. Full‐wave simulation results are summarized in Figure 9. Figure 9a,b present the LHCP phase and axial ratio (AR): four 90° phase states are realized at 3.75 GHz while maintaining AR < 3 dB. Figure 9c,d give the RHCP phase and AR, likewise achieving four 90° phase states at the center frequency with AR < 3 dB. These results confirm joint polarization conversion and 2‐bit phase coding.

**FIGURE 9:**
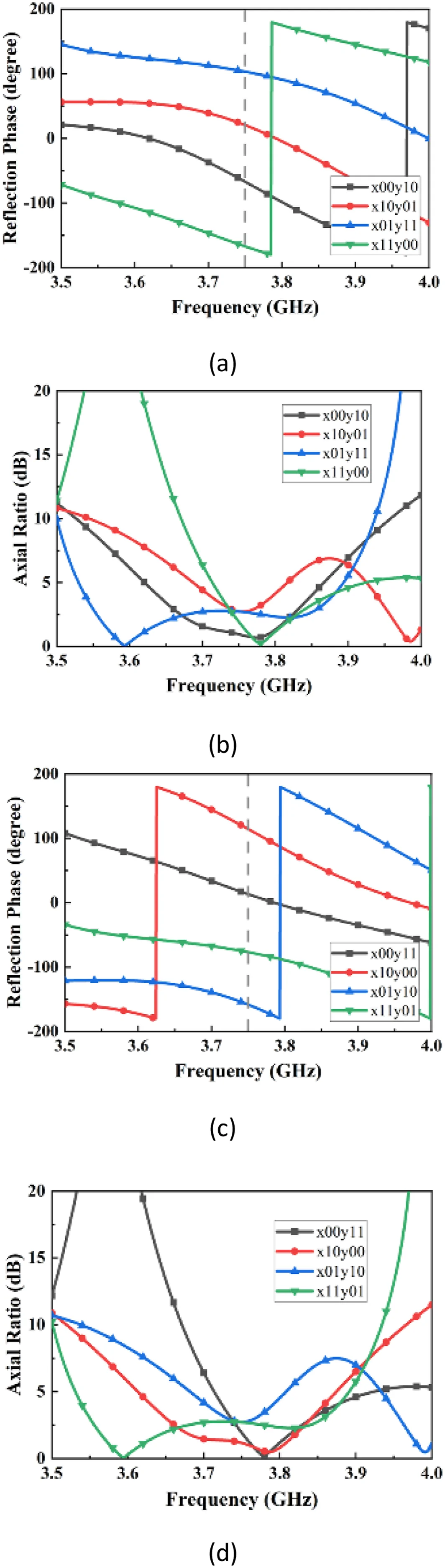
(a, b) reflected LHCP phase and axial ratio. (c, d) reflected RHCP phase and axial ratio.

### 3.2 Metasurface Design With Dynamic Functions

An 8 × 8 RRM is implemented. Each unit cell integrates six PIN diodes and is addressed by four independent bias lines, enabling 16 discrete tuning states per cell. The states are controlled by an external FPGA platform. The overall configuration is shown in Figure 10. When the reflective phase responses along the *x*‐ and *y*‐polarized components are kept identical, the RRM exhibits a uniform 2‐bit phase response for any incident polarization, thereby enabling polarization independent phase control over the half space. Moreover, imposing discrete linear phase gradients across the aperture steers the reflected beam to prescribed angles, whereas two‐dimensional phase layouts realize multidirectional beam pointing. Figure 11presents the coding matrices for different beam steering angles, along with the corresponding far‐field patterns. Figure 11a shows the coding matrix that synthesizes −10° deflection, whereas Figure 11c plots the resulting patterns for LP rotation angles of 0°, 30°, 60°, 90°, 120°, and 150°. Figure 11b depicts the coding matrix for −20° deflection, and Figure 11d reports the associated patterns under the same set of LP rotation angles.

**FIGURE 10:**
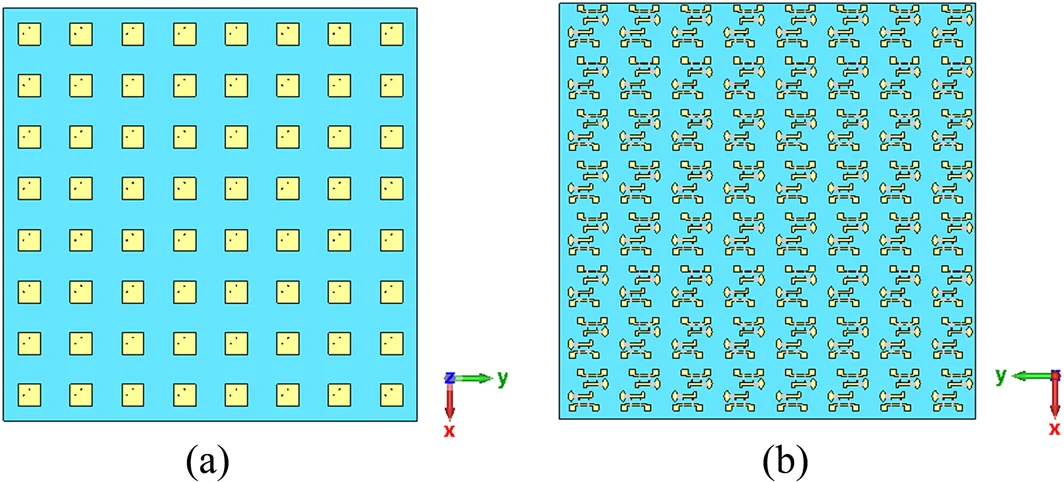
Array layout of the RRM. (a) The top view. (b) The bottom view.

**FIGURE 11:**
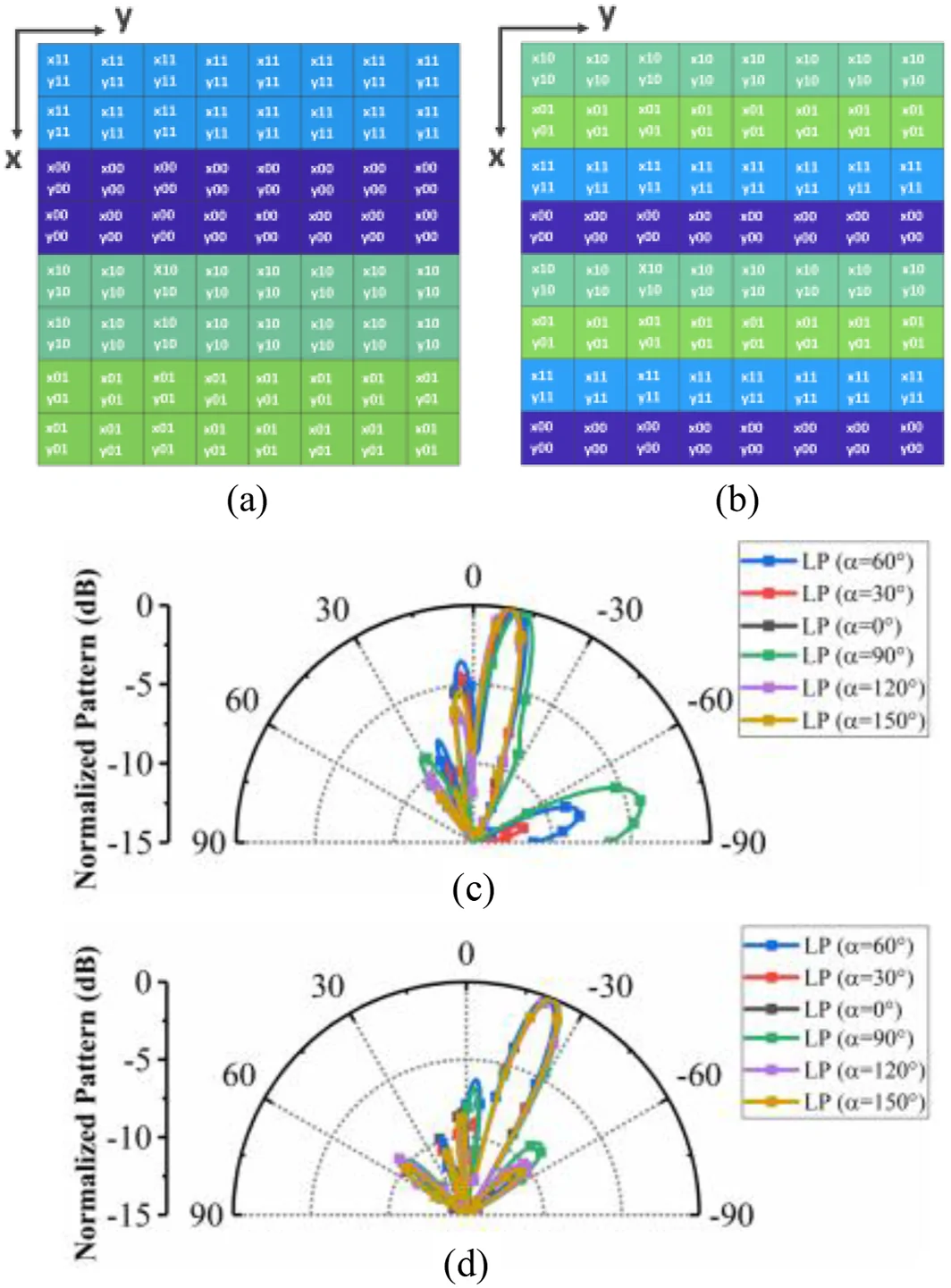
Coding matrices and far‐field patterns of the metasurface under LP incidence with different polarization rotation angles. (a) Coding matrix for −10° beam deflection. (b) Coding matrix for −20° beam deflection. (c) Far‐field patterns for −10° deflection at LP rotation angles of 0°, 30°, 60°, 90°, 120°, and 150°. (d) Far‐field patterns for −20° deflection under the same set of LP rotation angles.

Under a 45° LP incidence, the unit provides 16 discrete coding states, enabling 2‐bit reflected phase coding for polarization conversion: LHCP and RHCP with 0°, 90°, 180°, and 270° phase states available for each polarization. Figure 12 illustrates the coding patterns and far‐field radiation patterns for 20° beam deflection, confirming joint polarization conversion and beam shaping.

**FIGURE 12:**
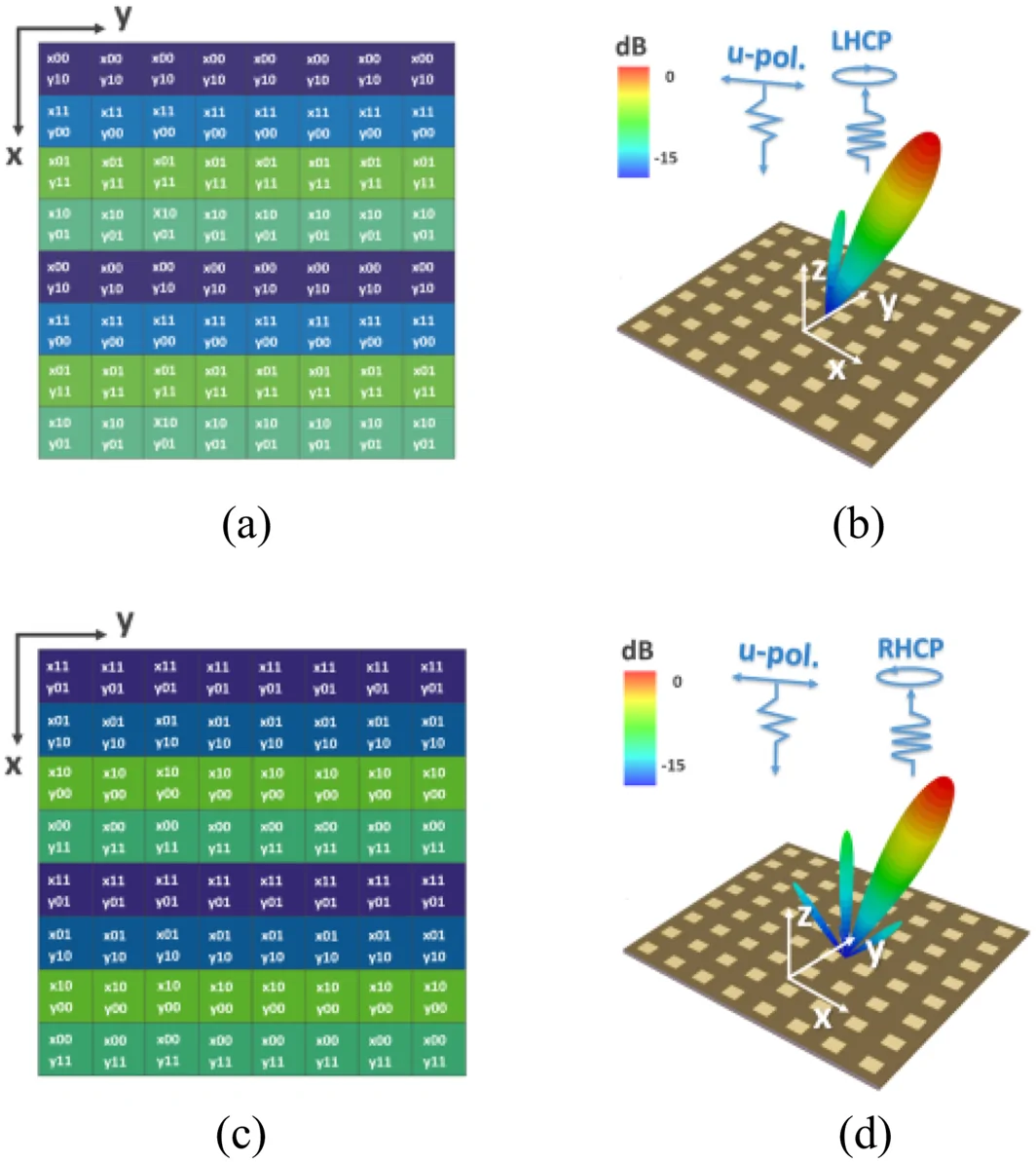
Metasurface under *u*‐polarized normal incidence with 20° beam deflection: coding matrices, far‐field patterns, and reflected polarization states for different conversions. (a) Coding matrix for LHCP reflection. (b) Far‐field pattern and polarization state for LHCP reflection. (c) Coding matrix for RHCP reflection. (d) Far‐field pattern and polarization state for RHCP reflection.

In addition, random phase coding over the aperture enables diffuse scattering and thus reduces the RCS. To enhance the RCS reduction, a Discrete Water Cycle Algorithm (DWCA) is employed to optimize the random phase distribution. The metasurface is configured according to the optimized coding matrix, achieving improved RCS reduction performance. Furthermore, Figure 13 compares the monostatic RCS of the metasurface and a reference PEC plate in the frequency range from 3.55 to 3.95 GHz at the observation angle (0°, 0°). In the band from 3.71 to 3.83 GHz, the monostatic RCS of the metasurface is reduced by more than 10 dB. These results demonstrate that the proposed 2‐bit metasurface provides excellent RCS reduction performance.

**FIGURE 13:**
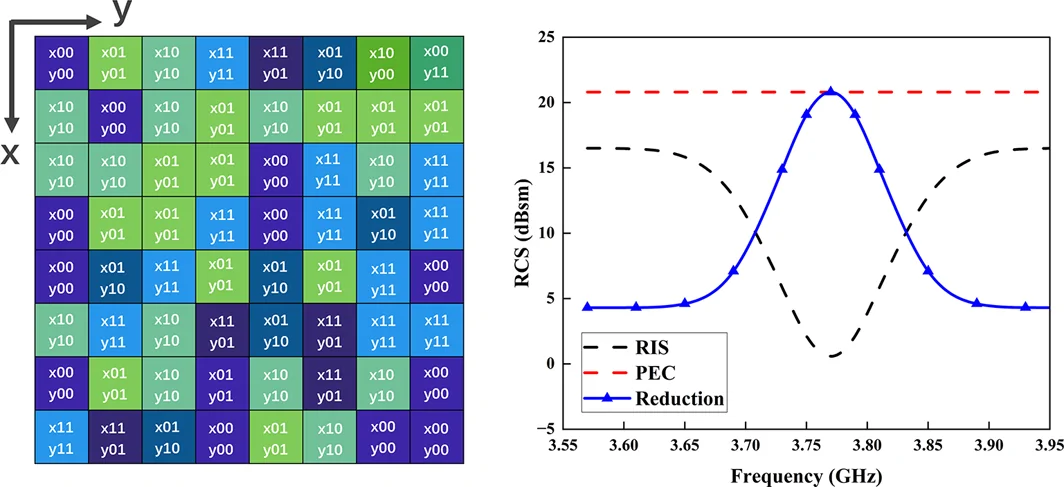
Monostatic RCS simulation results at (0°, 0°) of the proposed 2‐bit RRM under *y*‐polarized incidence. (a) Coding matrix for RCS reduction. (b) Comparison of the monostatic RCS between the metasurface and PEC.

## 4 Experimental Demonstration

### 4.1 Prototype and Fabrication

A 2‐bit RRM with an 8 × 8 element array was fabricated and tested. The aperture measures 460 × 560 mm, and the top layer is an 8 × 8 square patch array and each patch is via connected to the intermediate ground and the bottom phase shiFting layer, as shown in Figure 14a. In Figure 14b, the bottom layer integrates 8 × 8 reflective 2‐bit phase shiFters and each element is driven by four DC bias lines, yielding 256 bias channels routed to the array edges for ribbon connection. For real‐time control, the RRM is driven by an external FPGA board. The board provides four 68‐pin connectors and on each connector, pins 33, 34, 67, and 68 are GND, whereas the remaining 64 pins are general purpose GPIO, totaling 256 channels. The GPIO logic level is ± 2.5 V, and the 256 GPIO lines map to the 256 bias pads to enable per element addressing. To accelerate switching, coding matrices are preloaded into the FPGA memory, and the host communicates with the board via USB.

**FIGURE 14:**
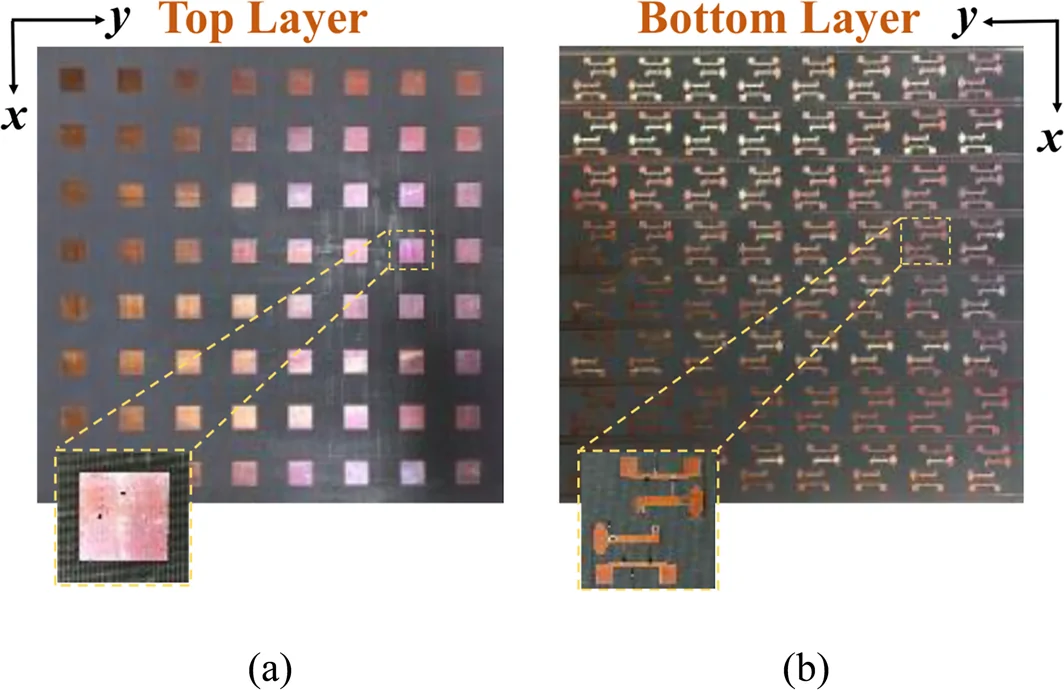
Photograph of the fabricated 2‐bit RRM prototype. (a) Top view of RRM. (b) Bottom view of RRM.

### 4.2 Experiments for the BCI‐Controlled Reconfigurable Metasurface

The experiment is carried out in an anechoic chamber using a vector network analyzer (VNA), an FPGA control board with a power supply, and two linearly polarized horn antennas. The RRM is on a low loss foam column with a motorized azimuth stage, as shown in Figure 15. Real‐time control of the 2‐bit RRM is evaluated. The Tx horn and the RRM are mounted on a motorized azimuth stage whose rotation axis is aligned with the surface normal. The polarization angle *α* of the incident field is set by rotating the Tx horn on a dedicated polarization rotation bracket.

**FIGURE 15:**
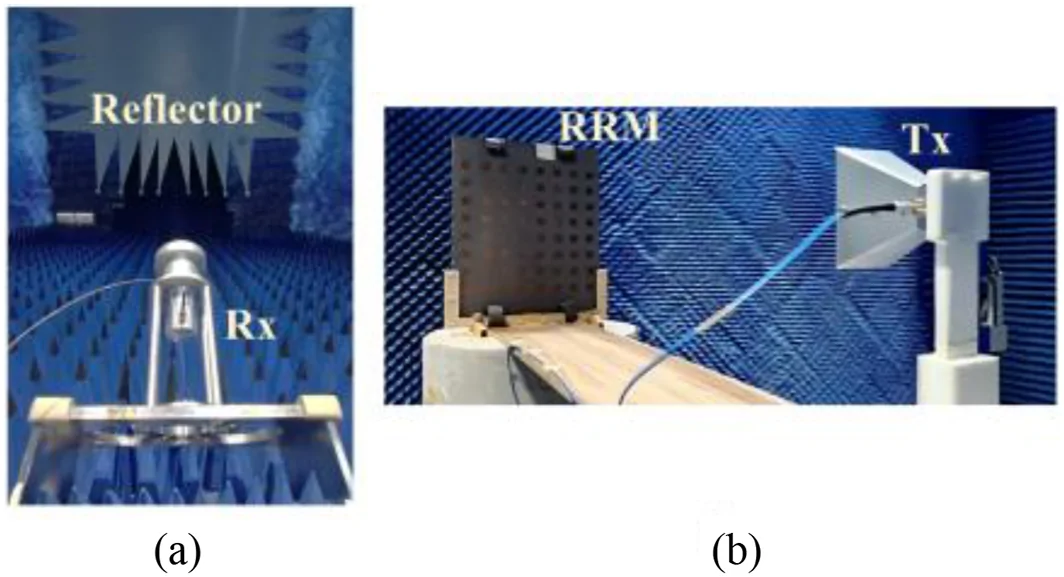
Photograph of the measurement setup. (a) Measurement setup, including reflector, transmit antenna, and receiver antenna. (b) Measurement setup, including RRM and FPGA.

The Rx horn is placed in the far‐field and the Tx‐RRM distance is about 1.2 m so that the illumination is in the far‐field and approximates a plane wave. The RRM is connected to an external FPGA via flat cables, and during measurements, the FPGA sequentially loads the phase‐coding matrices. The VNA records S21 across 3–5 GHz, whereas the rotary stage scans in 1° steps. Figure 16a presents the measured radiation patterns for incident LP angles of 30°, all showing a main beam steered to −10° at 3.75 GHz. The measured main beam consistently points to −10° with very small deviations. The 3 dB beamwidth agrees with the simulation within 2°. Figure 16b reports the −20° case. The measured main beam direction stays at target direction with a small deviation, which agree well with the expected results within very small deviations.

**FIGURE 16:**
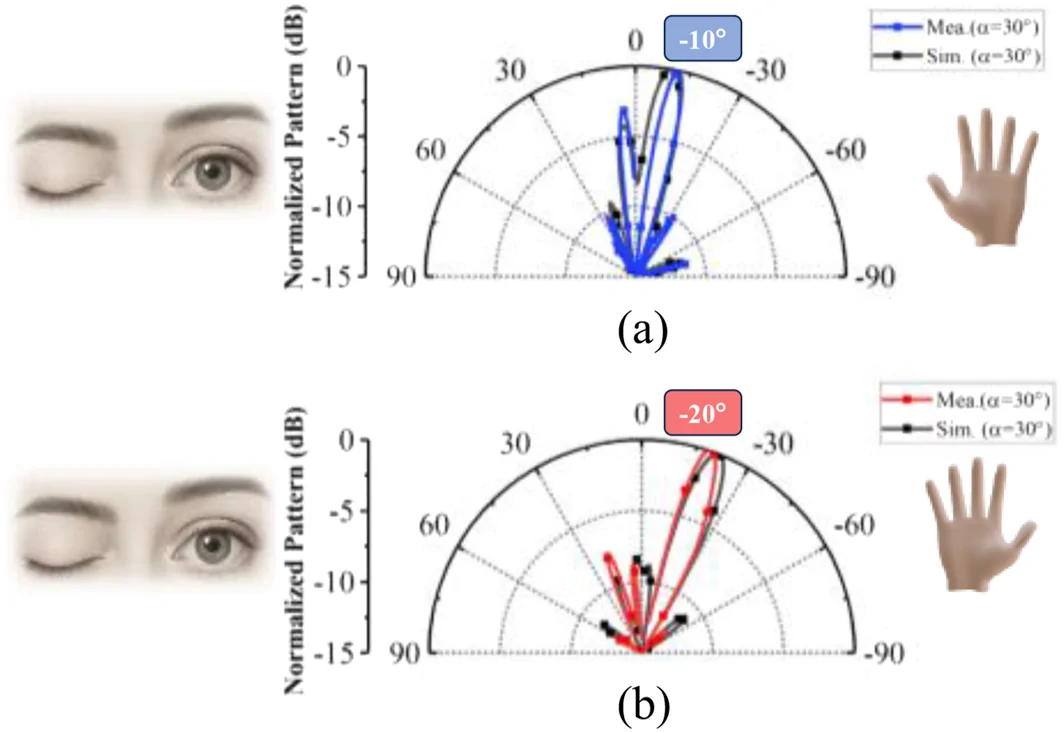
Results of Mode‐I. (a) −10° and (b) −20° steering performance at 3.75 GHz: comparison between measured and simulated normalized radiation patterns for polarization angles *α* = 30°.

Figure 17 verifies the joint polarization conversion and beam shaping under a 45° LP incident wave. Two reflected polarization states are tested. For demonstration, by performing a right‐eye blink and motor imagery of the left and right hands, the reflected wave achieves +20° beam deflection while simultaneously completing the polarization conversion. Across two polarization conversions, the main beam pointing remains +20° within small deviations. Figure 18 presents the RCS reduction measurement for Mode‐III. A reduction greater than 10 dB is achieved over 3.70–3.83 GHz.

**FIGURE 17:**
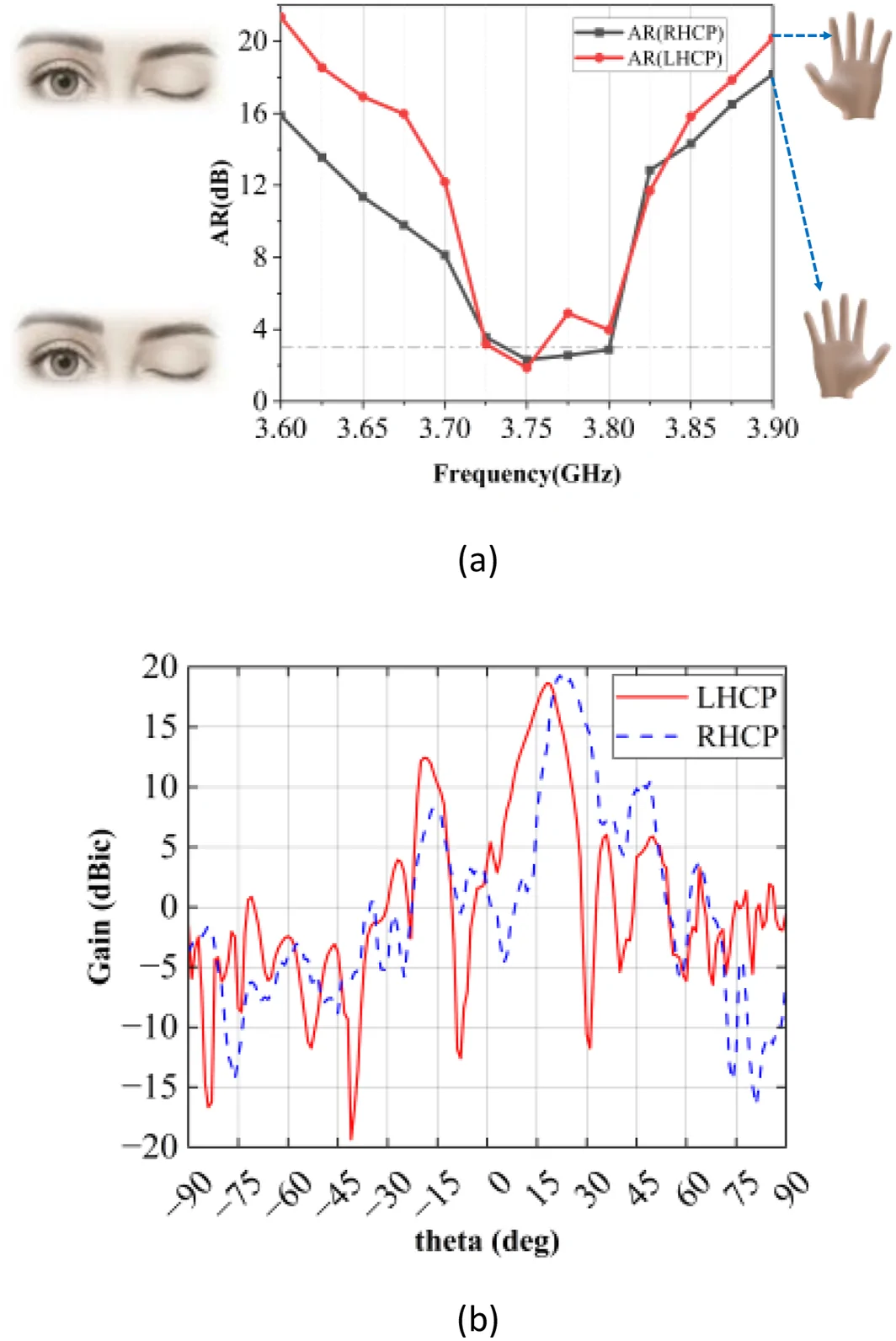
Results of Mode‐II circular polarization performance under BCI control. (a) Measured AR versus frequency for RHCP and LHCP; The right‐eye blink selects the CP state, and the hand motor‐imagery command triggers the operation. (b) Measured far‐field gain patterns of LHCP and RHCP at 3.75 GHz, showing the steered main lobe.

**FIGURE 18:**
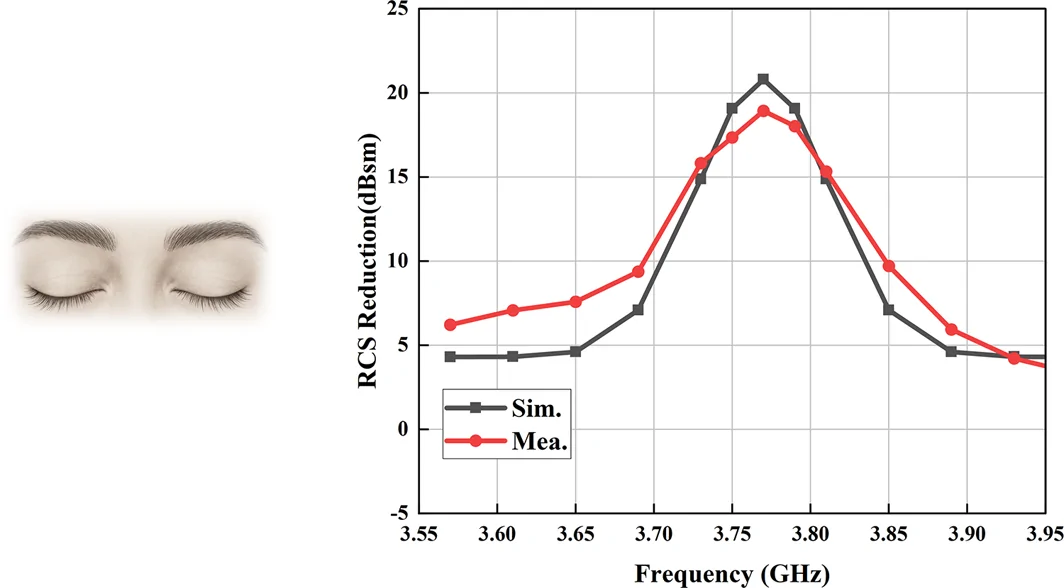
Mode‐III RCS reduction performance.

To validate the real‐time performance of the system, the test platform in Figure 19 comprises an EEG cap, a computer, an FPGA board, a signal generator, three horn antennas, and a digital oscilloscope. A 3.75 GHz continuous wave signal is radiated by the central transmitting horn to form a plane wave normally incident on the metasurface. Two receiving horns, Rx1 and Rx2, are fixed at the theoretical steering angles of +20° and −20°, respectively, and are connected to the oscilloscope to simultaneously record the time‐domain voltage envelopes in real time. The user first performs a left‐eye blink to enter the beam‐steering mode, then executes right‐hand motor imagery to trigger the phase‐coding command that steers the beam to −20°; correspondingly, the Rx2 trace increases markedly, whereas Rx1 falls to the noise condition. These complementary responses demonstrate real‐time, selective main‐lobe switching according to user intent. Because the oscilloscope provides only time‐domain waveforms rather than full radiation patterns, a fixed‐angle comparison is adopted in lieu of full‐angle scanning; the observed deflection closely matches the simulated target, confirming directional control of the brain‐controlled reconfigurable metasurface.

**FIGURE 19:**
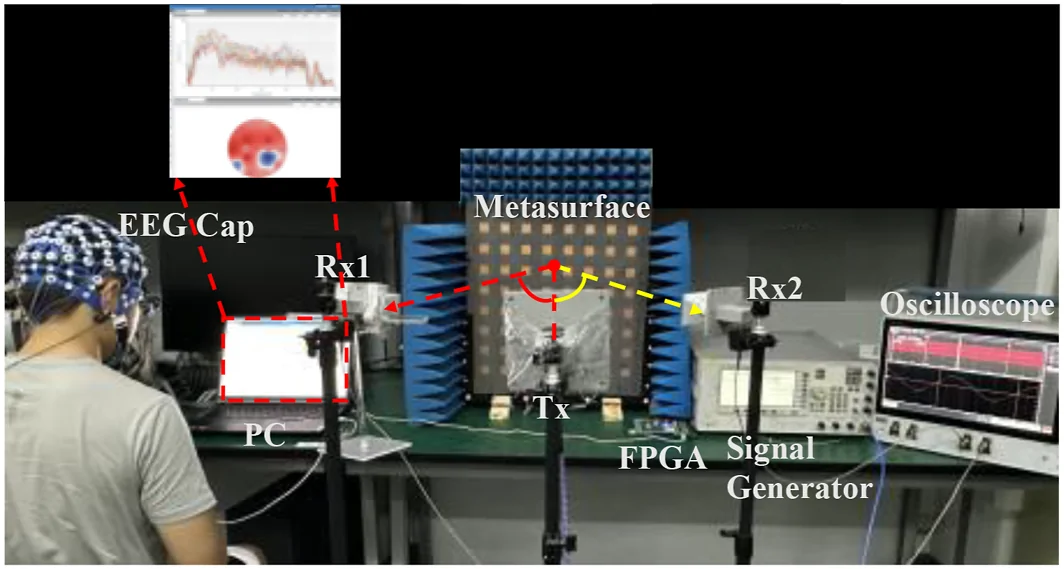
Real‐time system validation.

The total system latency is defined as the time required from the experimenter initiating the motor imagery command to the hypersurface reaching the updated command state, with an overall magnitude of approximately 3–5 s. The real‐time performance of the proposed system is primarily determined by the neural‐command generation and decoding process. The dominant latency originates from motor‐imagery execution and EEG signal accumulation, which are required to ensure reliable command recognition. In contrast, the computational latency of the MSFBCNN, FPGA command transmission, and metasurface state updating are comparatively small and contribute only a minor portion of the overall response time.

Consequently, the current system is suitable for mode switching and adaptive electromagnetic‐wave manipulation, where command updates occur on the order of seconds. Future improvements may be achieved through shorter decoding windows, more efficient neural‐decoding algorithms, and optimized signal‐processing pipelines to further reduce end‐to‐end latency.

## 5 Discussion

This study shows that coupling EOG‐triggered mode selection with MI–EEG based command refinement on a dual‐polarization 2‐bit reflective metasurface enables reliable, real‐time electromagnetic wave control across three complementary functions. Mode‐I delivers polarization‐agnostic 2‐bit reflection phase control. In Mode‐II, it achieves joint steering and polarization conversion under 45° LP incidence and Mode‐III achieves RCS reduction. The 8 × 8 prototype translates neural intents into discrete phase codes through a CNN‐to‐FPGA pipeline and achieves the measured steering and RCS outcomes summarized in the abstract, confirming the end‐to‐end feasibility of the proposed architecture. Relative to prior BCI demonstrations that actuate devices indirectly or metasurface studies that rely on scripted electronics, the present system establishes a direct link from neural activity to field manipulation without redesigning the metasurface hardware, whereas EOG‐based mode selection reduces inadvertent switching before MI–EEG commands are executed. Furthermore, extending the platform toward integrated sensing feedback, multiuser access, and task‐oriented objectives could further broaden its utility as a scalable neural interface for reconfigurable intelligent surfaces and reflectarray antennas.

It is worth noting that the reported 68%–74% accuracy corresponds to the more challenging LOSO cross‐subject evaluation. When training and testing are performed on the same subject, the classification accuracy reaches 79%–83%, indicating the potential of the proposed framework under personalized calibration settings. The reported LOSO accuracy of 68%–74% reflects the challenging nature of cross‐subject motor‐imagery EEG decoding. Although the achieved accuracy is sufficient for demonstrating proof‐of‐concept neural control of the metasurface, further improvements are desirable for practical large‐scale deployment. Performance differences among subjects suggest that intersubject physiological variability remains an important challenge for cross‐subject MI–EEG decoding. Variations in sensorimotor‐rhythm characteristics, attention levels, and individual neural activity patterns may affect decoding stability and contribute to the observed accuracy differences. A practical solution is to perform personalized calibration using a small amount of subject‐specific data to adapt the pretrained model to a new user.

Several approaches may be adopted to enhance decoding performance in future studies. Personalized calibration and subject‐adaptive learning can help reduce intersubject variability. Transfer‐learning techniques may be employed by pretraining models on large multisubject datasets and subsequently fine‐tuning them with a small amount of subject‐specific data. Domain‐adaptation methods may further improve cross‐subject generalization by aligning feature distributions among different users. In addition, data‐augmentation strategies and larger training datasets could further enhance model robustness and classification accuracy. Furthermore, hardware‐related improvements, such as increasing the number of EEG channels, improving electrode quality, and employing higher signal‐to‐noise‐ratio acquisition systems, may further enhance the quality of neural recordings and contribute to improved decoding performance.

The scalability of the proposed mapping strategy should also be considered for multidirection beam steering. In the current implementation, left‐hand and right‐hand MI are used as two directional control commands within the beam‐steering mode. A practical extension is to preserve the binary MI classifier and interpret the decoded commands as incremental steering operations rather than assigning each beam direction to an independent MI class.

For example, multiple steering states and their corresponding metasurface coding matrices can be preloaded in the FPGA. Assuming steering angles from −30° to +30° with a 10° step, the beam can be initialized at 0°. Consecutive left‐hand MI commands shiFt the beam toward + 10°, +20°, and + 30°, whereas consecutive right‐hand MI commands shiFt it toward −10°, −20°, and −30°. Boundary constraints can be applied to avoid invalid state transitions. With this strategy, the MSFBCNN output layer remains a two‐class soFtmax classifier, and the MI training‐data volume does not need to increase proportionally with the number of beam states. The main modifications are implemented in the command‐mapping logic and FPGA‐side beam‐state table, where additional coding matrices are preloaded and selected according to the decoded MI command.

Several factors may influence the performance and stability of the proposed brain‐controlled metasurface system. First, fluctuations in MI–EEG decoding accuracy may introduce occasional command errors due to intersubject variability, attention changes, and mental fatigue. Although the hierarchical EOG–EEG architecture reduces the risk of incorrect mode switching, decoding uncertainty may still affect the responsiveness and stability of task‐specific control.

Second, the electromagnetic performance of the metasurface may deviate from the ideal design due to several nonideal factors. Mutual coupling among adjacent unit cells may alter the intended phase distribution, whereas array‐factor‐related effects can lead to increased sidelobe levels during large‐angle beam steering. In addition, nonideal phase responses of reconfigurable unit cells may introduce discrepancies between theoretical coding matrices and measured beam directions or polarization characteristics. These effects become more pronounced as the functionality and reconfiguration complexity of the metasurface increase.

From an engineering implementation perspective, the large number of independently controlled elements requires a high‐density bias network, which may introduce parasitic coupling and signal crosstalk among bias lines. Variations in PIN‐diode switching characteristics, bias voltages, and device consistency may further affect the accuracy of the realized coding states. Moreover, the overall system relies on real‐time interaction among EEG acquisition, neural decoding, FPGA control, and metasurface reconfiguration, making synchronization and control consistency important practical considerations.

Finally, environmental factors such as electromagnetic interference and long‐term operational driFt may influence system performance in practical deployment scenarios. Future work will focus on improved electromagnetic modeling, optimized bias‐network design, adaptive calibration strategies, and more robust control architectures to further enhance system reliability and practical applicability.

## Author Contributions

G.‐M.Y. and X.W. conceived the study and supervised the project, X.H. completed the simulation and fabricated the samples, and completed the manuscript, R.S. participated in data analysis and experimental testing. All authors have accepted responsibility for the entire content of this manuscript and approved its submission.

## Funding

This work was supported in part by National Natural Science Foundation of China (Grants 62471146, 62401394), and in part by the State Key Laboratory of Radio Frequency Heterogeneous Integration Open Scientific Research Program (Grant KF2024017).

## Consent

Informed consent was obtained from all individuals included in this study.

## Conflicts of Interest

The authors declare no conflicts of interest.

## Data Availability

The datasets generated and/or analyzed during the current study are available from the corresponding author upon reasonable request.
